# Hypoxia inducible factors are dispensable for myeloid cell migration into the inflamed mouse eye

**DOI:** 10.1038/srep40830

**Published:** 2017-01-23

**Authors:** Peter J. Gardner, Sidath E. Liyanage, Enrico Cristante, Robert D. Sampson, Andrew D. Dick, Robin R. Ali, James W. Bainbridge

**Affiliations:** 1 UCL Institute of Ophthalmology, Genetics department, London, United Kingdom; 2 NIHR Biomedical Research Centre for Ophthalmology, Moorfields Eye Hospital, London, United Kingdom; 3 University of Bristol, Academic Unit of Ophthalmology, Bristol, United Kingdom

## Abstract

Hypoxia inducible factors (HIFs) are ubiquitously expressed transcription factors important for cell homeostasis during dynamic oxygen levels. Myeloid specific HIFs are crucial for aspects of myeloid cell function, including their ability to migrate into inflamed tissues during autoimmune disease. This contrasts with the concept that accumulation of myeloid cells at ischemic and hypoxic sites results from a lack of chemotactic responsiveness. Here we seek to address the role of HIFs in myeloid trafficking during inflammation in a mouse model of human uveitis. We show using mice with myeloid-specific Cre-deletion of HIFs that myeloid HIFs are dispensable for leukocyte migration into the inflamed eye. Myeloid-specific deletion of *Hif1a, Epas1*, or both together, had no impact on the number of myeloid cells migrating into the eye. Additionally, stabilization of HIF pathways via deletion of *Vhl* in myeloid cells had no impact on myeloid trafficking into the inflamed eye. Finally, we chemically induce hypoxemia via hemolytic anemia resulting in HIF stabilization within circulating leukocytes to demonstrate the dispensable role of HIFs in myeloid cell migration into the inflamed eye. These data suggest, contrary to previous reports, that HIF pathways in myeloid cells during inflammation and hypoxia are dispensable for myeloid cell tissue trafficking.

Myeloid cells play important roles in inflammation and autoimmunity. Here, the environment, including soluble factors present within tissues can modify myeloid cell phenotype and behavior, which in turn, can dictate the outcome of inflammation. For example, in rodent models of autoimmune uveitis, infiltrating myeloid cells display heterogeneous phenotypes throughout disease. Early on, they promote lymphocyte infiltration and drive retinal destruction through nitric oxide production^1^. In late-stage disease, myeloid cells can regulate pathology by suppressing T cell proliferation^2^, inhibiting T cell activation^3^,^4^, promoting the presence of regulatory T cells within the target organ^5^ and facilitating tissue repair^6^,^7^,^8^,^9^.

The hypoxia-inducible factor (HIF) pathway is integral for myeloid cell function and infiltration and was first described as a mechanism for sensing tissue hypoxia at the cellular level. In myeloid cells the HIF pathway comprises the alpha subunits HIF1α and HIF2α (encoded by *Hif1a* and *Epas1* respectively) both of which form heterodimers with HIF1β^10^. In normoxia, oxygen-dependent hydroxylases act on key proline residues on the alpha-subunits, allowing targeting of these proteins by the von Hippel-Lindau (VHL) E3 ubiquitin ligase complex for proteasomal degradation^11^,^12^. Conversely, in hypoxia this hydroxylation does not occur. The alpha-subunits accumulate in the cytoplasm, dimerize with the β counterpart subunits and subsequent nuclear translocation and transcription of downstream targets ensues^13^. Similarly, post-translational HIF stabilization has been demonstrated in innate inflammation^14^, in addition to transcriptional upregulation of *Hif1a* during normoxia in activated leukocytes^15^.

*In vitro* studies in conditional knockouts have shown that both HIF1α and HIF2α are essential for conventional myeloid function with *Hif1a* and *Epas1* deletion resulting in reduced phagocytosis, antigen presentation and bactericidal activity^15^,^16^,^17^. Likewise, stabilization of individual alpha subunits can polarize macrophages towards either an M1 or M2 phenotype, which is relevant to inflammation as M1-like macrophage-derived cytokines such as TNFα are central players in the pathogenesis of many chronic inflammatory and autoimmune diseases^18^.

However, the impact of the HIF pathway on myeloid cell migration and infiltration in inflammation remains unclear. While *in vitro* experiments demonstrate that *Hif1a*- and *Epas1*-deficient myeloid cells have a reduced potential to migrate in response to chemokines^19^,^20^, different *in vivo* disease models result in divergent phenotypes: a decrease in infiltrating myeloid cells is seen in cutaneous inflammation and an increase in the macrophage numbers in the kidney during renal inflammation when either *Hif1a* or *Epas1* is deleted^19^,^21^. Although infiltrating myeloid cells play key roles in ocular inflammation as outlined above, the importance of the HIF pathway within myeloid cells and its impact upon the kinetics of ocular inflammation remains unknown.

Noninfectious uveitis represents a broad spectrum of intraocular inflammatory conditions^22^. In man, noninfectious anterior uveitis (affecting the iris and ciliary body of the eye) is frequently acute and is associated with a wide range of systemic diseases including, spondyloarthritides, Behçet’s disease, inflammatory bowel disease and juvenile idiopathic arthritis^23^. Endotoxin-induced uveitis (EIU) in rodents, models aspects of human uveitis following delivery of lipopolysaccharide (LPS) into the vitreous^24^. In the mouse, it is characterized by an intraocular migration of myeloid cells from the blood, composed predominantly of neutrophils and inflammatory monocyte/macrophages. This myeloid infiltration can be enumerated by flow cytometry, peaking at 18 hours post induction and resolving with minimal tissue damage^25^. As LPS is a potent inducer of HIF stabilization^26^, we employed this model to investigate the importance of HIF pathways downstream of LPS induction on myeloid trafficking into inflamed ocular tissue in conditional knockout mice where HIF1a and HIF2a are either absent or stabilized in myeloid cells.

We report that neither *Lysm Cre*-driven myeloid-specific deletion of HIFs nor stabilization of HIF pathways has any significant effect upon myeloid numbers in the retina. Additionally, induction of hypoxia in peripheral blood (hypoxemia) using phenylhydrazine during EIU in myeloid-specific HIF deletion mice also had no significant effect upon myeloid infiltration, leading us to conclude that HIFs are dispensable for myeloid cell migration into the inflamed mouse eye, in contrast to other organs.

## Results

### Myeloid cell-specific Cre-mediated modulation of the HIF pathway does not influence myeloid infiltration into the eye during EIU

**Figure 1:**
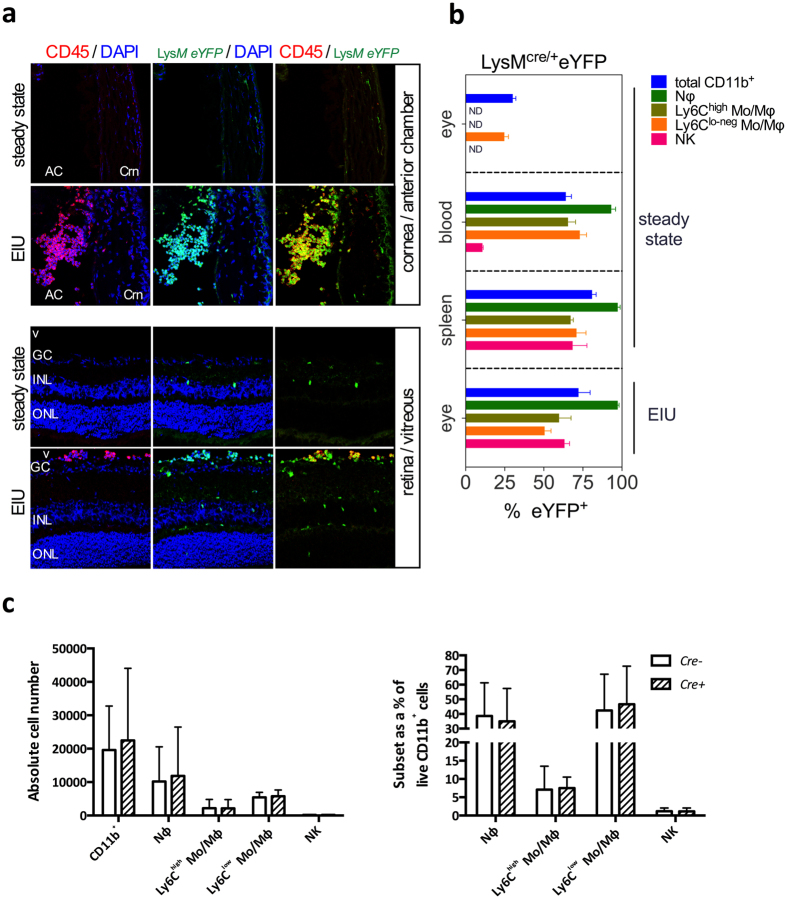
LysM-driven Cre expression occurs in EIU leukocyte infiltrate and has no impact on the number of infiltrating cells. (**A**) Confocal microscopy of ocular sections of *Lysm^eYFP^* mice in steady state and peak EIU showing eYFP^+^ (green) myeloid cells positive for CD45 (red) with a DAPI (blue) nuclear stain, Crn: Cornea, AC:Anterior chamber, GC:Ganglion cell layer, V:Vitreous, INL:Inner nuclear layer, ONL:Outer nuclear layer (all images 20× magnification). (**B**) Graphs showing the percentage of myeloid populations positive for eYFP from tissues of *Lysm^eYFP^* reporter mice in steady state and peak EIU (as specified), as quantified by flow cytometry; Graphs show mean ± SD; n = 4 per group (**C**) Graphs showing flow cytometric analyses of absolute cell numbers and proportions of various myeloid subsets infiltrated in the eye 18 hours after EIU induction in *Lysm^Cre/+^*  and wild type littermate controls. Myeloid cell populations are defined using standard gating strategy. Nϕ = neutrophils; Mo/Mϕ-monocyte/macrophages. Graphs show mean ± SD; n = 10–12 injected eyes per group; 3 independent experiments.

The myeloid infiltrate in EIU has been well characterized and is composed of predominantly neutrophils and inflammatory monocytes^25^. To confirm appropriate targeting of these infiltrating cells by *Lysm*-driven Cre recombinase (Cre) activity, we used confocal microscopy to analyse the anterior segment and retinal tissue from naïve and EIU-induced *Lysm*^+*/cre*^*Rosa26*^eYFP^ reporter mice. This showed that following EIU induction, there was an influx of eYFP^+^CD45^+^ cells into the anterior chamber, vitreous and retina of affected eyes when compared to the uninjected control eyes, where only a small number of resident myeloid cells (microglia) were labeled in the retina (Fig. 1a). Whilst the *Lysm* reporter activity within different myeloid cells has been reported previously^27^,^28^, the fidelity of *Lysm* expression within the infiltrating myeloid population in the eye during EIU is not known. We assessed this by flow cytometry using our recently published gating strategy^29^ (Supplemental Fig. 1) and observed a mean of 96% of CD11b^+^Ly6G^+^ neutrophils and a mean of 56% of CD11b^+^Ly6C^+^ inflammatory monocytes expressing eYFP in the eye at peak EIU; values which did not differ significantly from those observed in spleen and blood of steady state animals (Fig. 1b) and were comparable with those reported previously for spleen^27^ and similar to our previous findings in a mouse model of ocular neovascularization^28^. Interestingly, the mean percentage of CD11b^+^Ly6C^lo-neg^ cells expressing eYFP in the eye during peak EIU (49%) was significantly reduced compared to spleen (72%) and blood (70%) (Fig. 1b), consistent with reports that resident microglia express lower levels of *Lysm* compared to mature circulating myeloid cells^28^,^30^. NK cells can be differentiated from both lymphoid and myeloid progenitors^31^ and whilst not a major population infiltrating the eye during EIU^25^, we included them in the analysis due to their expression of CD11b.

The cellular production of the Cre recombinase has been reported to result in toxicity and altered phenotype in certain cell types^32^,^33^. To exclude this and the haploinsufficiency of the *Lysm* allele due to the knock-in of Cre^34^, we utilized flow cytometry to quantify the infiltrating myeloid subsets at peak disease, 18 hours post-EIU induction in *Lysm*^+*/Cre*^ mice and their wild type littermate controls. Comparison of both mean absolute numbers and subset percentages of the total CD11b^+^ myeloid population revealed no significant differences between groups (Fig. 1c).

**Figure 2:**
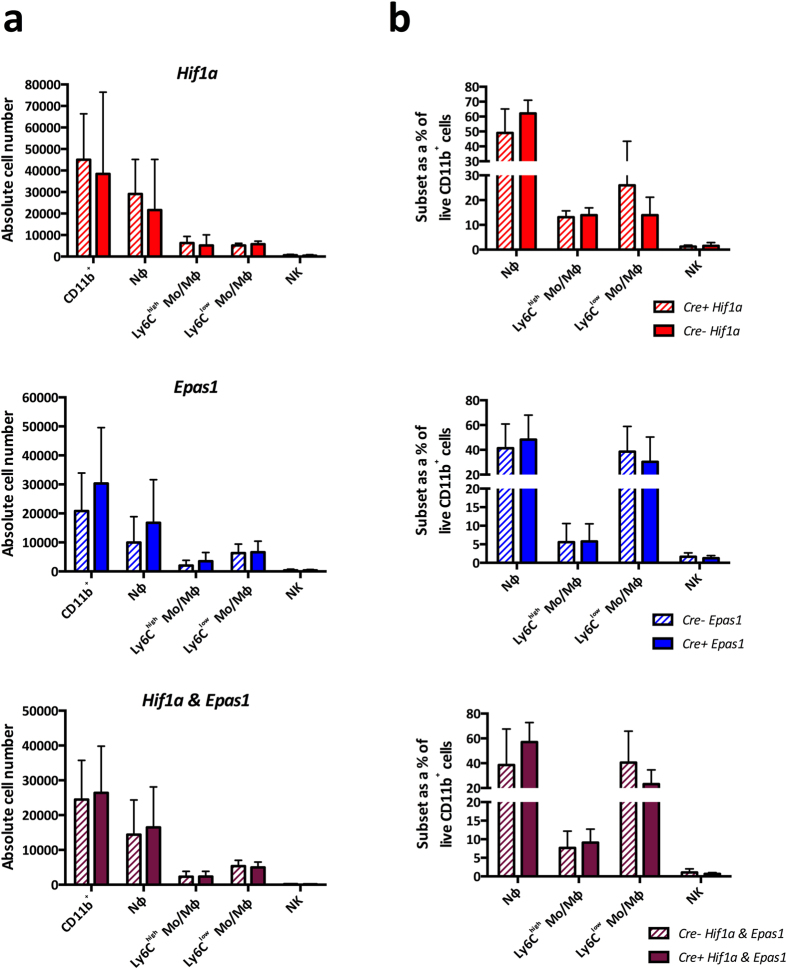
Deletion of HIF genes in myeloid cells does not influence EIU at peak disease. Flow cytometric analyses of (**A**) absolute cell numbers and (**B**) proportions of myeloid subsets infiltrated in the eye 18 hours after EIU induction in animals with myeloid cells deficient in *Hif1a, Epas1* and *Hif1a* & *Epas1* with their floxed littermate controls. Myeloid cell populations are defined using standard gating strategy. Nϕ = neutrophils; Mo/Mϕ-monocyte/macrophages. Graphs show mean ± SD; n = 10–12 injected eyes per group; > 3 independent experiments.

We next investigated the effect of deleting the HIF pathway within myeloid cells in EIU. We induced EIU in *Hif1a*^Δ/Δ^, *Epas1*^Δ/Δ^ and *Hif1a*^Δ/Δ^*Epas1*^Δ/Δ^ mice and their floxed littermate controls. In the mutants, Cre-mediated recombination results in the deletion of *Hif1a, Epas1*, or both *Hif1a* and *Epas1* respectively^28^. Analysis of the mean absolute numbers (Fig. 2a) and percentages (Fig. 2b) of myeloid cell populations infiltrating the eye at peak EIU showed no significant differences between any of the mutants and their controls. It has been reported that *Epas1* decreases neutrophil apoptosis in mice, resulting in persistent LPS-induced lung inflammation which fails to resolve^35^. To explore this and any impact on the HIF deletion on the resolution phase of EIU, we analyzed subset numbers and percentages in *Hif1a*^Δ/Δ^ and *Epas1*^Δ/Δ^ mutants at 48 hours post-EIU, finding no significant effect on the presence of myeloid cells when compared to controls (Supplemental Fig. 2A and B).

**Figure 3:**
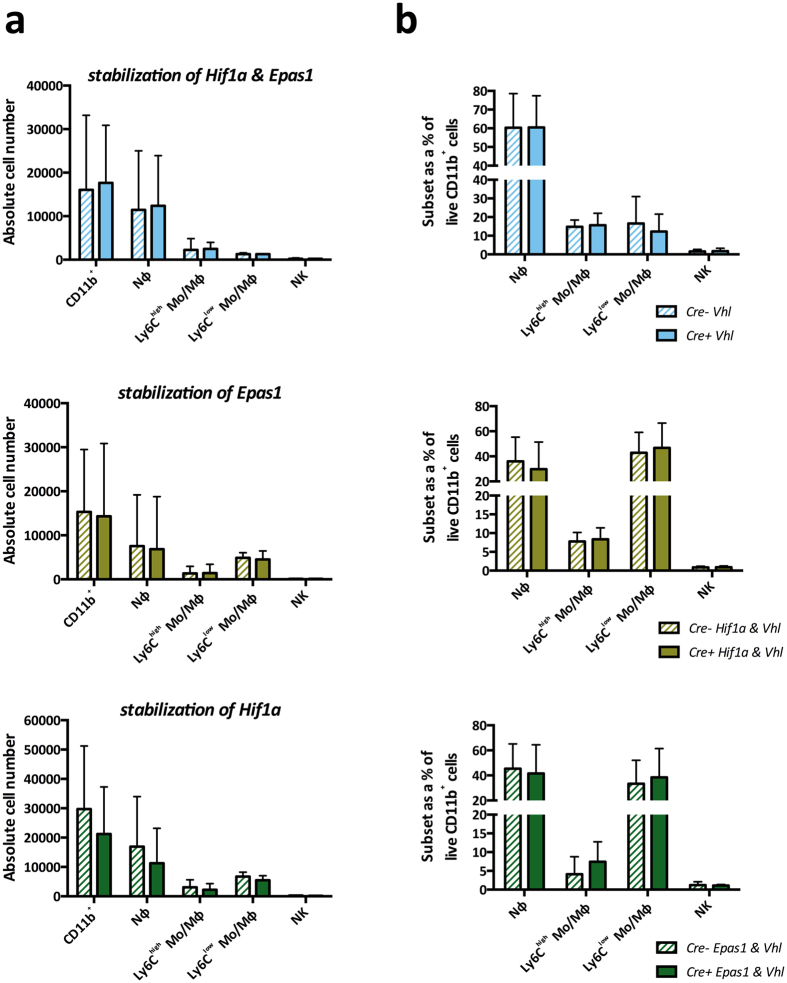
Stabilization of HIF proteins in myeloid cells does not influence EIU at peak disease. Flow cytometric analyses of (**A**) absolute cell numbers and (**B**) proportions of various myeloid subsets infiltrated in the eye 18 hours after EIU induction in animals with myeloid cells deficient in Vhl (stabilization of Hif1a and Epas1), Hif1a & Vhl (stabilization of Epas1) and Epas1 & Vhl (stabilization of Hif1a) with their floxed littermate controls. Myeloid cell populations are defined using standard gating strategy. Nϕ = neutrophils; Mo/Mϕ-monocyte/macrophages. Graphs show mean ± SD; n = 10–12 injected eyes per group; >3 independent experiments.

The Von Hippel-Lindau (VHL) protein, targets HIF proteins for proteasomal degradation during normoxia, and VHL deletion results in both HIF1α and HIF2α protein stabilization and pathway activation^12^. We used *Vhl*^Δ/Δ^*Epas1*^Δ/Δ^, *Vhl*^Δ/Δ^*Hif1a*^Δ/Δ^ and *Vhl*^Δ/Δ^ mice to stabilize *Hif1a, Epas1*, or both *Hif1a* and *Epas1* respectively. EIU induction in these and floxed littermate controls revealed that HIF stabilization did not affect myeloid trafficking into the inflamed eye at peak disease, in terms of either mean absolute numbers (Fig. 3a) or subset percentages (Fig. 3b).

### Phenylhydrazine administration results in hypoxemia and HIF stabilization in circulating leukocytes

While LPS is a potent inducer of HIF stabilization in leukocytes^26^,^36^, the focused nature of intraocular administration of LPS to induce EIU raised the possibility that HIF protein accumulation in response to LPS only occurs in resident retinal microglia, obscuring a direct effect upon leukocyte migration into the eye. To address this concern specifically given the low proportion (49%) of CD11b + Ly6C^lo-neg^ cells expressing eYFP in *Lysm*^+*/cre*^*Rosa26*^eYFP^ reporter mice at peak EIU we rendered the circulation hypoxic (hypoxemia) by chemically inducing hemolytic anemia, with consequent HIF stabilization within circulating leukocytes.

**Figure 4:**
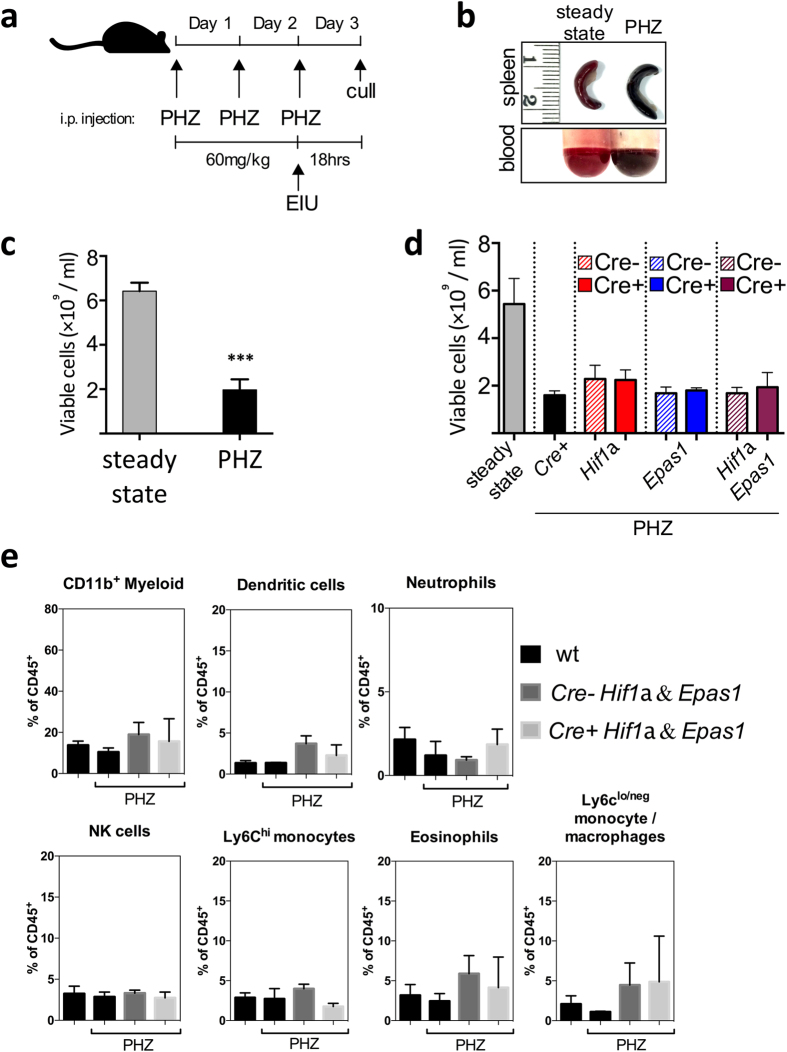
Phenylhydrazine (PHZ) administration results in hemolytic anemia and hypoxemia. (**A**) Schematic showing the PHZ dosing regimen; (**B**) Photographs of spleens and un-clotted blood from steady state mice and PHZ treated mice (**C**) quantification of viable cells in peripheral whole blood from mice treated with PHZ compared to untreated mice; (**D**) quantification of viable cells in peripheral whole blood from the various HIF-deletion mice treated with PHZ; (**E**) Flow cytometric analyses of PBMC purified by centrifugation through Histopaque-1083 with myeloid cell populations defined using standard gating strategy from mice untreated and treated with PHZ; Graphs show mean ± SD; n = 4 animals per group.

Phenylhydrazine (PHZ) causes red blood cell destruction by hemoglobin denaturation and lipid peroxidation^37^,^38^. PHZ administration to mice over a 48-hour period (Fig. 4a) resulted in features consistent with hemolytic anemia/hypoxemia such as splenomegaly and visibly dark red, deoxygenated blood (Fig. 4b) that have been previously reported^39^. PHZ administration reduced counts of viable peripheral blood mononuclear cells (PBMCs) by approximately 66% compared to untreated mice (Fig. 4c). A similar reduced level of viable PBMCs across all mouse mutants was observed following PHZ administration (Fig. 4d) and no significant impact on myeloid cell percentages in the blood was observed in *Hif1a*^Δ/Δ^*Epas1*^Δ/Δ^ mice and their floxed littermate controls following PHZ administration (Fig. 4e).

**Figure 5:**
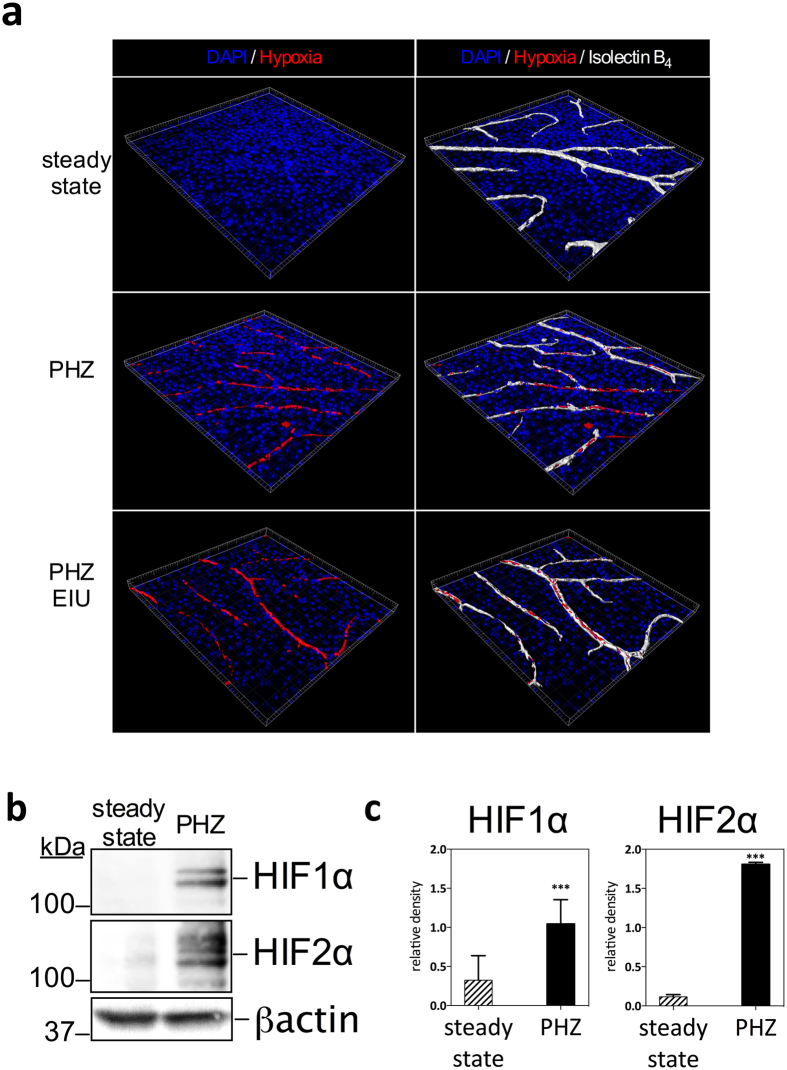
Phenylhydrazine administration results in hypoxia in retinal vessels and in HIF-1α and HIF-2α stabilization in peripheral blood leukocytes. (**A**) 3-dimensional reconstructed imaging of the superficial vascular plexus of flat mounted retinae of mice (steady state, treated with PHZ and treated with PHZ and peak EIU induction) stained with DAPI, hypoxyprobe and Isolectin B4. (**B**) Representative analysis of HIFs by western blot: 50ug of protein from total cell lysate of blood leukocytes isolated from mice treated with PHZ compared to untreated; (**C**) relative densitometry quantification of HIF western blots from PBMC showing mean, n = 3, error bar showing SD, unpaired t test, ***P < 0.001.

Analysis of the retinal vasculature of PHZ treated mice by flat mount staining following *in vivo* labeling with hypoxyprobe revealed that cells within retinal blood vessels in the superficial plexus stained positive for hypoxia compared with no staining in untreated steady state animals (Fig. 5a) or PHZ treated but hypoxyprobe unstained animals (no anti-hypoxyprobe antibody) and PHZ hypoxyprobe labeled animals but with the competed stain (Supplemental Fig. 2). The addition of peak EIU in PHZ treated animals resulted in similar hypoxia staining in retinal superficial plexus vessels (Fig. 5a). To confirm the validity of the PHZ-driven hypoxemia in activating HIF pathways in circulating leukocytes, we examined the stabilization of HIF1α and HIF2α proteins in blood isolated leukocytes by western blot. In steady state normoxic conditions, we could detect very little HIF1α or HIF2α protein (Fig. 5b) whereas in PHZ-treated animals we observed significantly increased levels of both proteins (Fig. 5b and c), indicating HIF stabilization.

### Myeloid-derived HIF1α and HIF2α are dispensable for myeloid cell trafficking into the eye during EIU

**Figure 6:**
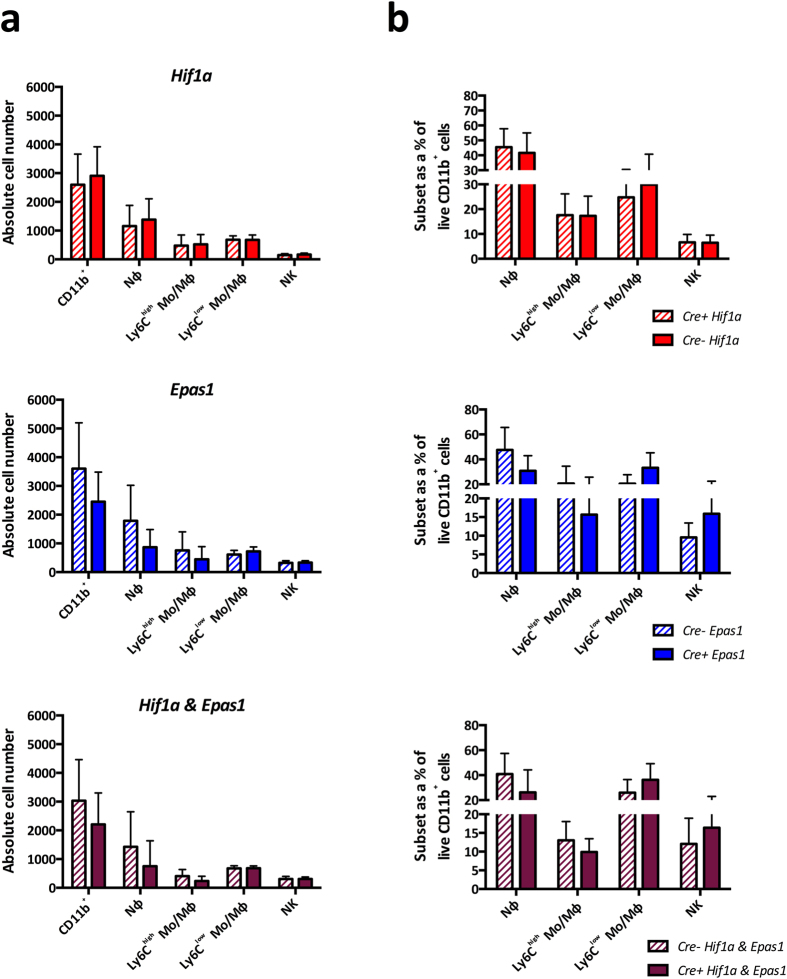
Deletion of HIF target genes in myeloid cells does not influence EIU at peak disease in the presence of hypoxia. Flow cytometric analyses of (**A**) absolute cell numbers and (**B**) proportions of various myeloid subsets infiltrated in the eye 18 hours after EIU induction in hypoxic animals with myeloid cells deficient in *Hif1a, Epas1* and *Hif1a* & *Epas1* with their floxed littermate controls. Myeloid cell populations are defined using standard gating strategy. Nϕ = neutrophils; Mo/Mϕ-monocyte/macrophages. Graphs show mean ± SD; n = 10–12 injected eyes per group.

The administration of PHZ in the regimen described results in hypoxic stabilization of the HIF pathway in circulating leukocytes. Inducing EIU in myeloid HIF-deficient animals on this background allowed us to further explore the role of HIF pathways in myeloid cell migration. The induction of hypoxemia via hemolytic anemia reduced the total number of viable cells in the blood stream largely through hemolysis, which resulted in a 10-fold reduced migration of leukocytes into the eye during concomitant EIU, that was consistent across all genotypes of mice studied. Analysis of the mean absolute numbers (Fig. 6a) and percentages (Fig. 6b) of myeloid cell populations infiltrating the eyes of PHZ treated peak EIU disease mice showed no significant differences between either *Hif1a*^Δ/Δ^, *Epas1*^Δ/Δ^ or *Hif1a*^Δ/Δ^*Epas1*^Δ/Δ^ mutants and their controls, implying that HIF pathways are not required for infiltration of myeloid cells into the eye during EIU.

## Discussion

Myeloid cells such as monocytes/macrophages and neutrophils are crucial early players in the immune response to infection and are known to carry out pivotal roles in the pathogenesis of autoimmune inflammatory disease^5^,^40^,^41^. It is therefore important to elucidate the mechanisms by which myeloid cells migrate to and are retained, at sites of inflammation. Myeloid cell infiltration into target tissues must be finely controlled during the acute inflammatory phase to enable resolution and avoid chronic inflammatory disease^42^. Control depends, in part, upon myeloid cell intrinsic responses to physiological cues such as the chemokine milieu^43^, the presence of apoptotic or necrotic cells^44^ and hypoxia^45^,^46^,^47^.

Studies of the HIF pathway in myeloid cells have so far resulted in a lack of consensus regarding the requirement of intact HIFs for migration^19^,^21^,^35^,^48^. Macrophages deficient in prolyl hydroxylases (PHDs) that target HIFs for degradation via VHL activity, exhibited increased chemotaxis *in vitro*^49^. Myeloid cells lacking HIF2α do not express M-CSF receptor, abolishing recruitment of cells via this chemokine^20^ and HIF2α null neutrophils show reduced infiltration during lung inflammation^35^. HIF1α null macrophages have a decreased capacity for migration both *in vitro* and *in vivo*^19^. This was thought to be due to HIF1α driving the switch from aerobic respiration to anaerobic glycolysis during low oxygen levels, thereby increasing the intracellular pool of ATP necessary for cellular trafficking in the hypoxic microenvironment^19^,^50^,^51^. However a recent report has highlighted that HIF1α null macrophages have a total ATP content greater than wild type cells, suggesting that their deficiency in infiltration is independent of cellular energy levels^48^.

Importantly, evidence also suggests a lack of HIF pathway involvement in myeloid migration, as HIF2α null macrophages show no defect in migration^48^. Additionally, hypoxia can act to retain myeloid cells within both tumors and inflamed tissues. This is thought to occur via limitation of myeloid responses to MCP-1 and the CCR5 ligands MIP1α and MIP1β, resulting in myeloid cell residency within hypoxic sites^52^,^53^,^54^,^55^. It is unclear then, whether HIF pathways act to enhance or limit myeloid tissue infiltration during inflammation. General aspects of macrophage aggregation, motility and infiltration have been shown reliant on HIF pathways, with impaired homotypic adhesion (HA) observed in HIF1α null macrophages^19^. Leukocyte HA relies on LFA-1/ICAM-1 and VLA-4/VCAM-1 interactions^56^,^57^ and blocking both of these in mice suppresses the ocular infiltrate during EIU^58^,^59^ highlighting EIU as an appropriate model for investigating the importance of HIF pathways in myeloid migration.

We sought to elucidate the impact of myeloid-intrinsic HIFs on myeloid cell trafficking into the inflamed eye by effective manipulation of the HIF pathway using Cre-recombinase mediated deletion driven by the *Lysm* promoter. The EIU stimulus, LPS, within the immune privileged ocular environment results in local production in the eye of various cytokines and chemokines including those relevant to the HIF pathway: MCP-1, MIP1α and MIP1β^25^, although production of M-CSF has not been reported. The subsequent myeloid infiltration from the circulating blood can be accurately quantified using multi-colour flow cytometry^29^ and biological effects observed^25^. The absence of a quantifiable phenotype in mutants where HIF alpha-subunits were either deleted or stabilized suggests no involvement of HIF pathways in myeloid migration in response to LPS.

EIU was induced in ‘knockout-reporters’, where Cre-mediated recombination results in the deletion of *Hif* genes and production of GFP. Analysis of infiltrating myeloid cells showed that the percentage of GFP^+^ cells was not significantly different to controls for neutrophils and inflammatory monocytes in the HIF1α- and EPAS1-deficient mice (Supplemental Fig. 3). While the fidelity of this reporting system may not be 100%, these data suggest that selection of HIF-deficient myeloid cells is unlikely to occur in EIU as *Hif* gene knock out cells are present in the eye. Lysm expression in monocyte/macrophage cells is partial in blood and spleen including the eye and this is an important caveat when considering our data and that already published with these mice. Lysm expression in monocyte/macrophage cells is partial in blood and spleen including the eye and this is an important caveat when considering our data and that already published with these mice^15^,^19^,^20^,^21^,^26^,^35^,^60^. It is possible that there may be redundancy provided by LPS-induced chemokines signaling outside of HIF pathways, masking a myeloid migration phenotype to specific chemokines. However, work with MCP-1/CCR2 deficient mice revealed that the loss of MCP-1 alone severely curtails the ocular infiltrate in EIU^25^,^61^. Importantly, our findings contrast with previous *in vitro* and *in vivo* studies utilizing models where significant tissue hypoxia exists (cutaneous inflammation) or where oxygen tensions can vary widely (lung)^19^,^20^,^62^. It is uncertain whether significant HIF induction in myeloid cells occurs during EIU and it is possible that the normoxic milieu in EIU may limit the requirement for HIF1α to drive active processes of migration into the eye. We therefore refined our model to investigate this by inducing EIU in HIF-deficient animals with concomitant hypoxemia. As hypoxia is the canonical inducer of the HIF pathway, inducing hypoxemia by PHZ treatment (with the subsequent stabilization of HIF proteins) allowed us to specifically explore the requirement of HIFs for inflammatory trafficking of myeloid cells from a hypoxic environment into the inflamed eye.

PHZ administration resulted in a general suppression of myeloid cells infiltrating the eye during EIU by approximately 10 fold across all HIF deletion mice and controls, with no difference in all myeloid subsets examined. This suggests a general effect on both HIF-deficient and wild type cells following erythrocyte hemolysis and no specific impact of PHZ on mutant cells. Despite the induction of the HIF pathway by concomitant hypoxemia during EIU, we observed no effect upon migrating numbers of myeloid cells into the eye at peak disease in animals devoid of myeloid-derived HIF, when compared to controls. This suggests that HIF alpha subunits are not required for the infiltration of myeloid cells into the eye. Again, this finding contrasts with previous *in vivo* studies in the same mouse mutants that show either decreased or increased numbers of inflammatory cells in cutaneous^19^ and renal inflammation^21^. Importantly, recent studies using cutaneous and renal inflammatory mouse models in the same mutant mice report contrasting data with those above; i.e. normal myeloid infiltration following specific myeloid deletion of HIF1α^15^,^63^. Whilst the supposition is that stimuli strength may dictate the role of HIF1α in myeloid cells^15^, it is feasible that different trafficking phenotypes observed in myeloid-specific HIF deletion mice are model-specific, depending on the tissue, disease-kinetic and experimental method employed.

Therefore, our use of hypoxemia during EIU allowed us to investigate the impact of HIF pathways in myeloid cells when migrating from the hypoxemic circulation into an inflamed tissue devoid of apparent hypoxia. In doing so, we demonstrate that HIF pathways are dispensable for myeloid migration from a hypoxic environment into the inflamed eye. In the wider context, our findings may apply to diseases that exhibit hypoxemia and inflammation, such as chronic obstructive pulmonary disease^64^ and sickle cell disease^65^. Our findings also challenge currently held concepts that chemokine insensitivity explains the accumulation of myeloid cells at ischemic and hypoxic sites^66^.

Importantly, we did not explore *in vivo* myeloid function *per se* in terms of myeloid-specific cytokine/chemokine production and neutrophil apoptosis (both shown to be affected by HIF pathway deficiencies), as EIU is a sterile model of inflammation with minimal tissue damage and is therefore not suitable to address these issues. However, the benefit of our approach lies in the use of an *in vivo* model of leukocyte migration into the inflamed eye, a relatively contained organ, from which accurate cell counts can be obtained. We highlight a specific model of ocular inflammation in which the HIF pathway is dispensable for myeloid migration from the hypoxic blood into the eye.

These findings not only inform about the role of HIF pathways within myeloid cells, but also relate to those seeking to manipulate HIFs therapeutically for the control of myeloid infiltration during inflammation. Importantly, we highlight the requirement of multiple and varied *in vivo* studies in elucidating downstream effects of the HIF system within myeloid cells in specific tissues.

## Methods

### Animals

All mouse procedures were conducted under the regulation of the UK Home Office Animals (Scientific Procedures) Act 1986, with University College London ethics committee approval and in compliance with the Association for Research in Vision and Ophthalmology (ARVO) statement for the Use of Animals in Ophthalmology and Vision Research. The following mice were used: *Lysm*^Cre/+^ crossed with B6.Cg-*Gt(ROSA)26Sor*^*tm3(CAG-EYFP)Hze*^/J, *Lysm*^Cre/+^*Hif1a*^flox/flox^, *Lysm*^Cre/+^*Epas1*^flox/flox^, *Lysm*^Cre/+^*Hif1a*^flox/flox^*Epas1*^flox/flox^, *Lysm*^Cre/+^*Vhl*^flox/flox^, *Lysm*^Cre/+^*Vhl*^flox/flox^*Hif1a*^flox/flox^, *Lysm*^Cre/+^*Vhl*^flox/flox^*Epas1*^flox/flox^. ‘Knockout-reporters’ included *Lysm*^Cre/+^, *Lysm*^Cre/+^*Hif1a*^flox/flox^ and *Lysm*^Cre/+^*Epas1*^flox/flox^ crossed with B6.*Gt(ROSA)26Sor*^*tm4(ACTB-tdTomato,-EGFP)Luo*^/J. Adult male and female mice of all experimental genotypes used in this study were compared to age matched littermate controls.

### Endotoxin induced uveitis (EIU) induction

Disease was induced following induction of general anesthesia using ketamine by administration of 1ng of LPS dissolved in phosphate buffered saline (PBS) by intravitreal injection of 2 μl using a microsurgical syringe and 38 gauge needle (Hamilton, Switzerland).

### Induction of hemolytic anemia

Phenylhydrazine hydrochloride from Sigma (cat #114715) was dissolved in PBS to make a 12 mg/ml solution. Mice were treated by 100 ul i.p. injections at 60 mg/kg doses according o the regimen detailed in Fig. 4a^39^.

### Immunohistochemistry and Fluorescent imaging

Eyes were enucleated and fixed in 4% paraformaldehyde (PFA) for 1 hour before freezing in OCT medium (Fisher Scientific, UK) and 16-μm cryosections cut. Sections were blocked in 5% donkey serum for 1 hour prior to staining with rat anti-mouse CD45 (MCA1388, AbD serotec) and secondary goat anti-mouse IgG Alexa Fluor 546 (A11081, Life Technologies) prior to imaging with a Leica DM5500Q confocal microscope.

### Flow cytometry

Enucleated eyes were dissected in 100 μL of cold Dulbecco’s modified Eagle’s media. After incision at the limbus with a 29-gauge needle, a circumferential cut around the eye following the limbus was made. Iris was dissected away, releasing anterior chamber infiltrating cells into the dissection media. The retina and vitreous were then removed carefully from the eye-cup, leaving the sclera/retinal pigment epithelium/choroid intact. The dissection media containing anterior fluid, vitreous, and retina were then pipetted up into a 1.5-mL Eppendorf tube and mechanically disrupted by rapping 10 times for a single-cell suspension, followed by centrifugation through a single well of a 96-well, 60-mm cell strainer plate (Millipore, Watford, UK). The resulting single cell suspensions were blocked with anti mouse CD16/32 (2.4G2) (eBioscience) for 5 minutes prior to surface staining with the following antibodies: CD11b (M1/70) BV711 (Biolegend), CD11c (N418)FITC (eBioscience), Ly6G (1A8)BV421(Biolegend), NK1.1 (PK136)PerCP/Cy5.5 (Biolegend), Ly6C (HK1.4)BV510 (Biolegend), CD45 (clone) BUV395 (BD Biosciences). Absolute cell numbers were obtained as previously detailed^29^. As we observed no gross developmental difference in eye size of mutant mice compared to floxed control mice we therefore considered cell counts from each sample (retina and intraocular fluid) to be inherently normalized as the absolute number and comparable between samples. Counts were obtained using a BD LSR Fortessa-x20 using FACSDiva software 8.1 (BD Cytometry systems). Single-stained beads (OneComp eBeads; eBioscience) of all fluorochromes were used to generate compensation matrices and fluorescence-minus-one controls were used for positive gating; data were analysed using FlowJo software (Treestar, USA). Cells were defined with the following stains: live, CD45^+^ singlets were gated CD11b^+^ for total myeloid; CD11b^+^CD11c^+^ for dendritic cells; CD11b^+^Ly6G^+^ for neutrophils, CD11b^+^NK1.1^+^ for NK cells; CD11b^+^Ly6C^hi^ for inflammatory monocytes; CD11b^+^Ly6C^lo/neg^ for monocytes/macrophages; and CD11b^+^Ly6C^int^SSC^hi^ for eosinophils^29^.

### Flat mount hypoxyprobe staining

An i.p. injection of 200 μl hypoxyprobe (EF5; 10 mM in 0.9% saline solution; University of Pennsylvania, USA) was administered to the mouse 12 and 2 hours prior to culling. Eyes were enucleated and fixed for 2 hours in 4% paraformaldehyde (PFA). Retina whole mounts to be stained were incubated at room temperature (RT) for 1 hour in freshly prepared blocking buffer (5% normal goat serum; 1% BSA; 1% Triton X-100 in PBS 1X) and subsequently overnight at 4 °C with biotin-conjugated isolectin B4 (1:200 in blocking buffer; Sigma-Aldrich) to stain for retinal vasculature. Tissue was washed 3 × 15 mins in washing buffer (1:1 blocking buffer in PBS 1X), incubated in AF633-conjugated streptavidin (1:500 in blocking buffer; Sigma-Aldrich) for 2 hours at RT and then washed again 3 × 15 mins in 0.3% Triton X-100 PBS 1X. Samples were then briefly fixed in 1% PFA for 10 mins at RT, incubated for 1 hour at RT in blocking buffer and incubated overnight at 4 °C in Cy3-conjugated ELK-351 antibody (diluted 1:1 in blocking buffer). Staining controls (one unstained and one competed stain) were also prepared *as per* manufacturer’s instructions. Whole mounts were then washed 2 × 45 mins in 0.3% Triton X-100 PBS 1X and 1 × 45 mns in PBS 1X. Following 10 mins incubation with Hoechst and 2 × 10 mins washes in PBS 1X, samples were mounted using mounting medium (Dako Ltd.) and a coverslip, with the ganglion cell layer facing upwards. Fluorescent images of the vascular superficial plexus were acquired on a confocal laser-scanning microscope (TCS SPE; Leica Microsystems (UK) Ltd., UK) using a 40X oil immersion objective and 3D reconstructed with Imaris software (Bitplane).

### Western blot

Detection of HIF1α and HIF2α was carried out on the total cell lysate from blood leukocytes. Leukocytes were rapidly purified from 500 ul of peripheral whole blood obtained by cardiac puncture (into EDTA Vacutainer tubes, BD Biosciences) by hypotonic erythrocyte lysis: 9 ml of water was added, immediately mixed followed by the addition of 1 ml of 10X PBS and centrifugation. The remaining leukocyte suspension was lysed RIPA buffer containing 1:100 freshly added protease inhibitor cocktail (Sigma, Gillingham, UK) with shaking for 20 min at 4 °C. Proteins were separated using 9% SDS-PAGE and blotted to Immobilon-P transfer membrane (Millipore, Watford, UK), followed by detection with anti-HIF1α (NB100–449, Novus, BioTechne), anti-HIF2α (NB100–122, Novus, BioTechne) antibodies, anti-Actin antibody as a loading control (ab1801, abcam, Cambridge, UK) followed by incubation with the secondary Goat anti-Rabbit-HRP and subsequent development with ECL Plus detection kit (Amersham, GE Healthcare, UK). Stripping buffer: 10% SDS, 60 mM Tris-HCL pH6.8, 300 mM *β*-Mercaptoethanol was used for 20 minutes at 45 °C between HIF1α and HIF2α antibody incubations/exposures.

### Cell counts

Viable cell counts from peripheral whole blood obtained by cardiac puncture (into EDTA Vacutainer tubes, BD Biosciences) were determined using an automated cell counter (Vi-CELL XR cell viability analyzer, Beckman Coulter (UK) Ltd.).

### Statistical analysis

Data were analyzed for statistical significance using Graphpad Prism software. Tests used are detailed either in the figure legends and P values for the EIU infiltrate analysis are in table form as Supplemental Table S1.

## Additional Information

**How to cite this article**: Gardner, P. J. *et al*. Hypoxia inducible factors are dispensable for myeloid cell migration into the inflamed mouse eye. *Sci. Rep.* **7**, 40830; doi: 10.1038/srep40830 (2017).

**Publisher's note:** Springer Nature remains neutral with regard to jurisdictional claims in published maps and institutional affiliations.

## Supplementary Material

Supplemental Figures
