# Supplementary material for: Hypoxia inducible factors are dispensable for myeloid cell migration into the inflamed mouse eye
Source: Sci Rep. 2017 Jan 23;7:40830. doi: 10.1038/srep40830 (PMC5256030; doi:10.1038/srep40830)
Supplement: Supplemental Figures [file srep40830-s1.pdf]

Hypoxia inducible factors are dispensable for myeloid cell migration into the inflamed mouse eye

Peter J Gardner[1*#], Sidath E Liyanage[1#], Enrico Cristante[1], Robert D Sampson[1], Andrew D Dick[1, 2, 3], Robin R Ali[1, 2] and James W Bainbridge[1, 2]

1 UCL Institute of Ophthalmology, Genetics department (London, (United Kingdom)
2 NIHR Biomedical Research Centre for Ophthalmology, Moorfields Eye Hospital (London, (United Kingdom)
3 University of Bristol, Academic Unit of Ophthalmology (Bristol, (United Kingdom)
# Authors contributed equally
*Corresponding author: Dr. Peter J Gardner email: p.gardner@ucl.ac.uk

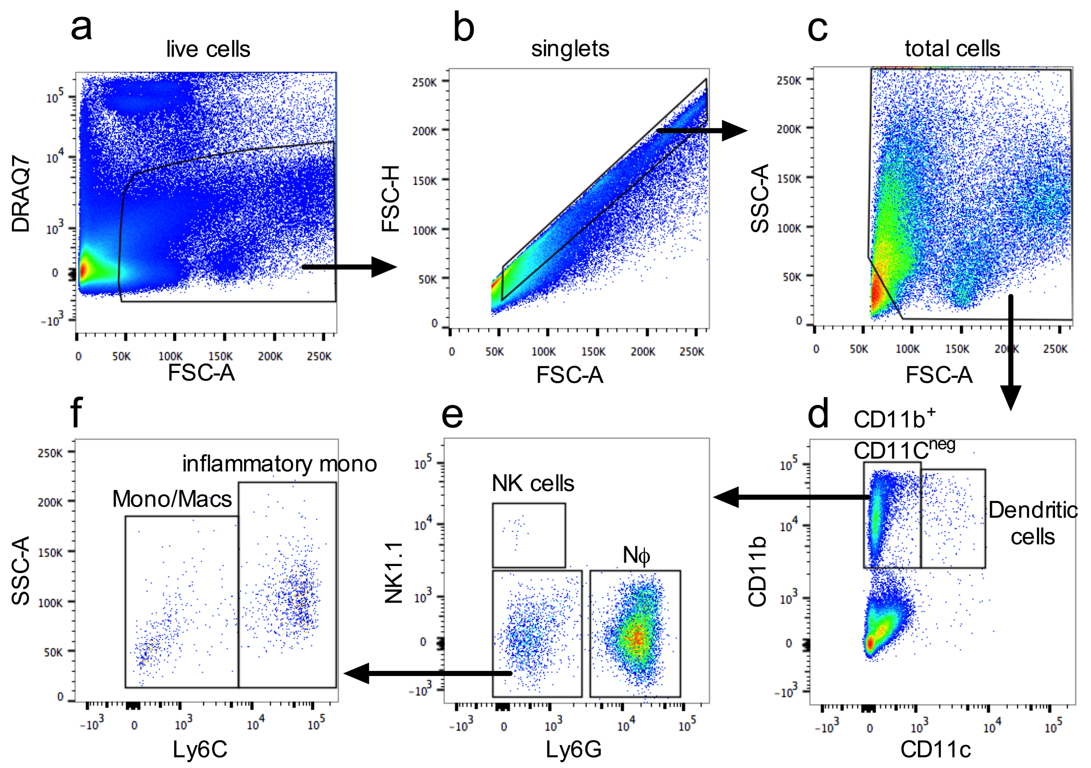

Supplemental Figure 1. Flow cytometry gating strategy. Flow cytometric gating strategy for myeloid cells in the mouse eye 18 hours after EIU induction in WT C57BL/6 mice; (a) live cells are gated using the DRAQ7 dye to stain dead cells (b) singlets are gated, (c) based on event size and granularity debris and non leukocytes are gated out, (d) myeloid cells are gated are gated away from retinal cells using CD11b but excluding $CD11c^{+}$ dendritic cells from analysis, (e) cells are gated for ($NK1.1^{+}$) Natural killer cells and ($Ly6G^{+}$) Neutrophils, (f) the remaining cells are gated using expression of Ly6C to discriminate ($Ly6C^{lo\text{-}neg}$) monocyte/macrophages and ($Ly6C^{hi}$) inflammatory monocytes.

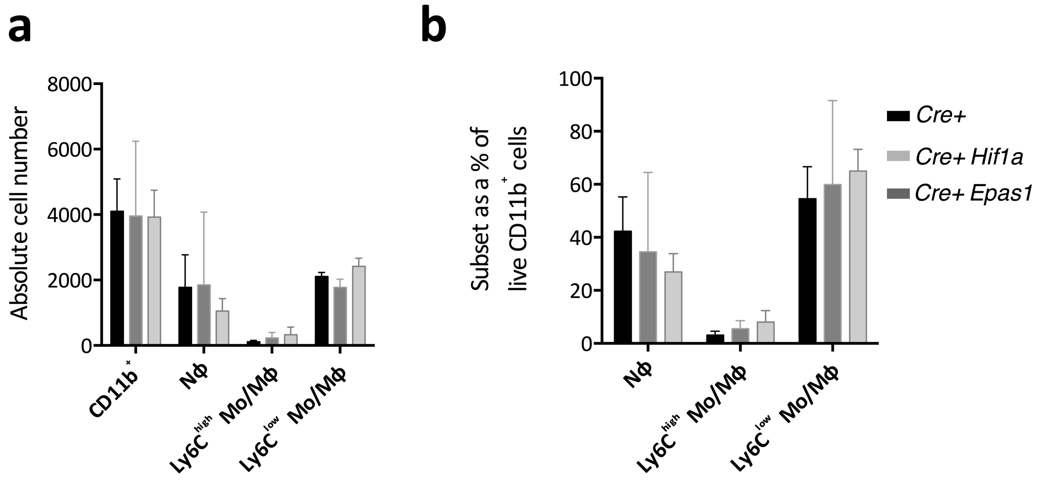

Supplemental Figure 2. Assessment of EIU at 48 hrs post induction. Flow cytometric analyses of (a) absolute cell numbers and (b) proportions of myeloid subsets infiltrated in the eye 48 hours after EIU induction in *Lysm*$^{Cre/+}$ animals and mice with myeloid cells deficient in *Hif1a* or *Epas1*: Myeloid cell populations are defined using standard gating strategy. Nϕ = neutrophils; Mo/Mϕ - monocyte/macrophages. Graphs show mean ± SD; n = 10 - 12 injected eyes per group.

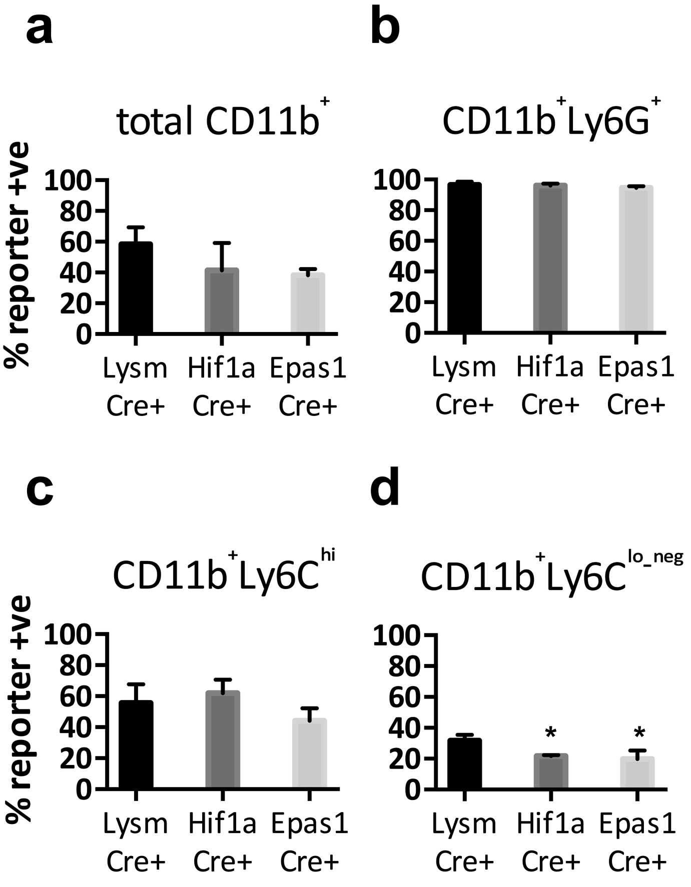

Supplemental Figure 3. Assessment of the presence of HIF knock-out cells in the eye during EIU. Flow cytometric analyses of GFP production driven by Lysm/Cre-mediated deletion of a floxed stop codon in floxed Hif1a and Epas1 mice. Data show the proportion of myeloid subsets positive for GFP infiltrating the eye during EIU as compared to *Lysm*$^{Cre/+}$ eYFP reporter animals, a) total CD11b^{+} myeloid;b) CD11b+Ly6G Neutrophils**;** c) CD11b+Ly6C^{hi}**;** d) CD11b+Ly6C$^{lo\text{-}neg}$. Myeloid cell populations are defined using standard gating strategy. Graphs show mean ± SD; n = 3 - 5 injected eyes per group, Kruskal-Wallis one-way ANOVA, * P=0.0165.

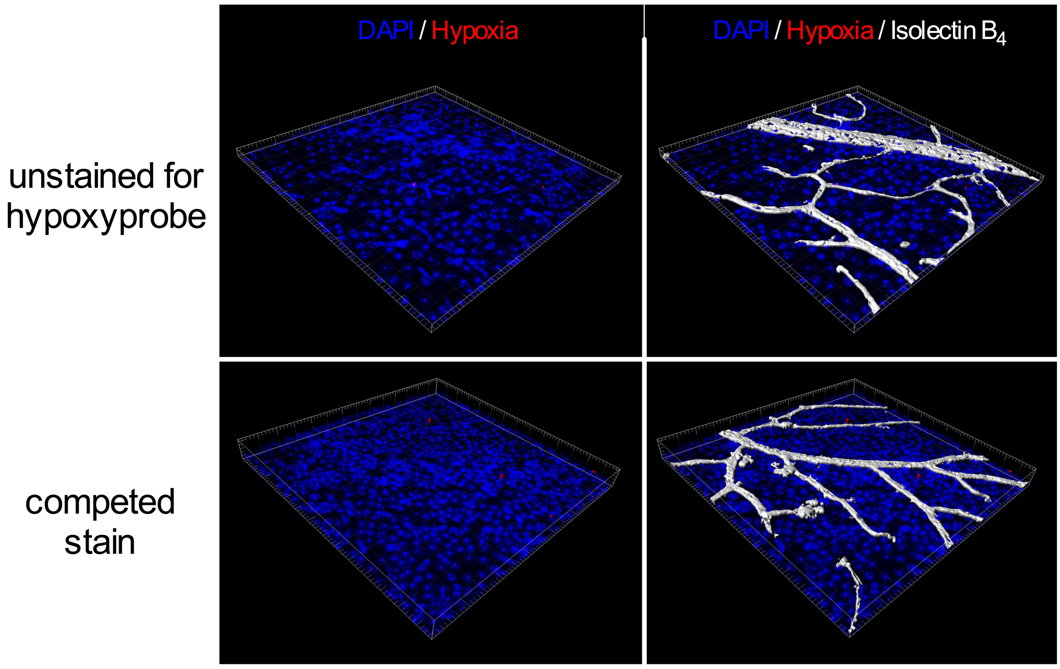

Supplemental Figure 4. Flat mount retina controls for hypoxia staining. 3-dimensional reconstructed imaging of superficial plexus from flat mounted retinae of PHZ treated mice either unstained for hypoxyprobe (no anti-hypoxyprobe antibody) or PHZ mice following two i.p. injections with hypoxiprobe 12 and 2 hrs prior to culling and Hypoxyprobe-competed stain and staining with DAPI, hypoxyprobe and Isolectin.

figure 1

		cell numbers		percentages
Cre	$CD11b^+$	0.697424		
	Nϕ	0.750487	Nϕ	0.696097
	$Ly6C^{high}$ Mo/Mϕ	0.970296	$Ly6C^{high}$ Mo/Mϕ	0.837179
	$Ly6C^{low}$ Mo/Mϕ	0.671288	$Ly6C^{low}$ Mo/Mϕ	0.687397
	NK	0.977758	NK	0.947649

figure 2

		cell numbers		percentages
Hif1a	$CD11b^+$	0.603547		
	Nϕ	0.376868	Nϕ	0.0217093
	$Ly6C^{high}$ Mo/Mϕ	0.521054	$Ly6C^{high}$ Mo/Mϕ	0.504333
	$Ly6C^{low}$ Mo/Mϕ	0.223981	$Ly6C^{low}$ Mo/Mϕ	0.0386536
	NK	0.349505	NK	0.50572
		cell numbers		percentages
Epas1	$CD11b^+$	0.0512081		
	Nϕ	0.0605257	Nϕ	0.231222
	$Ly6C^{high}$ Mo/Mϕ	0.0385813	$Ly6C^{high}$ Mo/Mϕ	0.905162
	$Ly6C^{low}$ Mo/Mϕ	0.774895	$Ly6C^{low}$ Mo/Mϕ	0.16498
	NK	0.977471	NK	0.175579
		cell numbers		percentages
Hif1a / Epas1	$CD11b^+$	0.718826		
	Nϕ	0.654876	Nϕ	0.0859047
	$Ly6C^{high}$ Mo/Mϕ	0.916377	$Ly6C^{high}$ Mo/Mϕ	0.428685
	$Ly6C^{low}$ Mo/Mϕ	0.597299	$Ly6C^{low}$ Mo/Mϕ	0.0588428
	NK	0.528388	NK	0.257931

figure 3

		cell numbers		percentages
Vhl	$CD11b^+$	0.815316		
	Nϕ	0.870075	Nϕ	0.990025
	$Ly6C^{high}$ Mo/Mϕ	0.820353	$Ly6C^{high}$ Mo/Mϕ	0.737409
	$Ly6C^{low}$ Mo/Mϕ	0.884046	$Ly6C^{low}$ Mo/Mϕ	0.435415
	NK	0.949043	NK	0.865256
		cell numbers		percentages
Hif1a / Vhl	CD11b+	0.873441		
	Nϕ	0.881888	Nϕ	0.467648
	$Ly6C^{high}$ Mo/Mϕ	0.940761	$Ly6C^{high}$ Mo/Mϕ	0.602551
	$Ly6C^{low}$ Mo/Mϕ	0.582693	$Ly6C^{low}$ Mo/Mϕ	0.595893
	NK	0.926561	NK	0.661085
		cell numbers		percentages
Epas1 / Vhl	$CD11b^+$	0.286565		
	Nϕ	0.357838	Nϕ	0.664635
	$Ly6C^{high}$ Mo/Mϕ	0.391069	$Ly6C^{high}$ Mo/Mϕ	0.120752
	$Ly6C^{low}$ Mo/Mϕ	0.0570904	$Ly6C^{low}$ Mo/Mϕ	0.557369
	NK	0.162802	NK	0.653947

figure 6

		cell numbers		percentages
Hif1a	$CD11b^+$	0.513827		
	Nϕ	0.493108	Nϕ	0.509409
	$Ly6C^{high}$ Mo/Mϕ	0.782881	$Ly6C^{high}$ Mo/Mϕ	0.946335
	$Ly6C^{low}$ Mo/Mϕ	0.923466	$Ly6C^{low}$ Mo/Mϕ	0.209387
	NK	0.387515	NK	0.928142
Epas1	CD11b+	0.071339		
	Nϕ	0.0612485	Nϕ	0.0313879
	$Ly6C^{high}$ Mo/Mϕ	0.227254	$Ly6C^{high}$ Mo/Mϕ	0.373304
	$Ly6C^{low}$ Mo/Mϕ	0.124648	$Ly6C^{low}$ Mo/Mϕ	0.0131476
	NK	0.685331	NK	0.0296188
Hif1a / Epas1	$CD11b^+$	0.164664		
	Nϕ	0.172859	Nϕ	0.074792
	$Ly6C^{high}$ Mo/Mϕ	0.0777661	$Ly6C^{high}$ Mo/Mϕ	0.123479
	$Ly6C^{low}$ Mo/Mϕ	0.827501	$Ly6C^{low}$ Mo/Mϕ	0.0671834
	NK	0.913782	NK	0.165067

Supplemental Table S1. P values from statistical analyses carried out on EIU infiltrate data. Absolute counts and myeloid subset percentages of total $CD11b^+$ cells were compared between mutant and floxed control mice as shown in Fig. 1, 2, 3 and 6, using multiple comparison t tests with statistical significance determined using the Holm-Sidak method with alpha =5.0%
